# From darkness to twilight: Morphological divergence between cave and surface‐subterranean ecotone *Niphargus* species

**DOI:** 10.1002/ece3.70061

**Published:** 2024-08-06

**Authors:** Anna Biró, Gergely Balázs, Žiga Fišer, Cene Fišer, Gergely Horváth, Gábor Herczeg

**Affiliations:** ^1^ Department of Systematic Zoology and Ecology Institute of Biology, ELTE Eötvös Loránd University Budapest Hungary; ^2^ HUN‐REN‐ELTE‐MTM Integrative Ecology Research Group Budapest Hungary; ^3^ Doctoral School of Biology Institute of Biology, ELTE Eötvös Loránd University Budapest Hungary; ^4^ Department of Biology, Biotechnical Faculty University of Ljubljana Ljubljana Slovenia

**Keywords:** adaptation, amphipod, convergent evolution, crustacean, divergent evolution, sexual dimorphism, springs, subterranean habitat

## Abstract

Subterranean and surface habitats are in stark contrast in several environmental factors. Therefore, adaptation to the subterranean environment typically impedes the (re)colonisation of surface habitats. The genus *Niphargus* includes amphipod crustaceans that primarily occupy subterranean habitats. All its species show typical adaptations to the subterranean environment. However, some *Niphargus* species occur in surface‐subterranean ecotones. To understand whether (i) habitat‐based phenotypic divergence is present between the cave and the ecotone species and (ii) similar phenotypes emerge independently in each ecotone, we studied morphological divergence between four cave and four ecotone *Niphargus* species based on 13 functional morphological traits. To account for different selection acting on the sexes, we included both males (*N* = 244) and females (*N* = 222). Nine out of 13 traits showed habitat‐divergence. Traits related to feeding and crawling were shorter, while traits related to oxygenation were larger in ecotone species. Eleven out of 13 traits were sexually dimorphic. Traits related to oxygenation and crawling were larger in females, while the trait related to swimming was larger in males. We found that the extent of sexual dimorphism differs between the habitats in eight traits related to sensing, feeding, oxygenation and crawling. Additionally, we found that in certain traits related to sensing and oxygenation, habitat‐related differences are only present in one sex, but not the other. We conclude that the detected differences between the cave and the ecotone species indicate divergent evolution, where similarities among the different species within habitat type indicate convergent evolution. The high degree of sexual dimorphism paired with differences in sexual dimorphism between the habitats in certain traits suggest that sexual and fecundity selections have comparable effects to environmental selection. Thus, studies of habitat‐dependent adaptations investigating one sex only, or not considering sexual dimorphism, can lead to erroneous conclusions.

## Graphical abstract

The genus *Niphargus* comprises species primarily dwelling in the subterranean environment, but some of them colonised surface‐subterranean ecotones. Comparing 13 morphological traits of cave‐ and ecotone‐dwelling species, we found strong habitat‐related divergence that also includes the extent of sexual dimorphism in certain cases. Our results suggest that an interplay among environmental, sexual and fecundity selection resulted in the observed patterns.

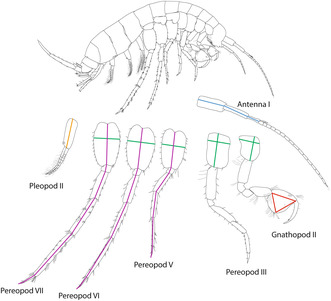

## 1 INTRODUCTION

Phenotypic variation in the wild results from various environmental and genetic factors (West‐Eberhard, 2003). A common driver of phenotypic evolution is natural selection. In ecologically heterogeneous environments, taxa tend to diverge into distinct phenotypes through divergent evolution (Fitzpatrick, 2012; Schluter, 2000), while in environments sharing selective regimes, phenotypes attain similarity through convergent or parallel evolution (Endler, 1986; McGhee, 2011). Besides environmental selection, natural selection also includes sexual and fecundity selection (Bonduriansky, 2001; Pincheira‐Donoso & Hunt, 2017). Sexual and fecundity selection can contradict environmental selection, favour different adaptive optima for females and males, and lead to sexual dimorphism (Butler et al., 2007; da Silva et al., 2014; Fairbairn et al., 2007; Pincheira‐Donoso & Hunt, 2017). Hence, phenotypic variation in the wild is a result of phenotype performance in survival or resource‐competition, along with fecundity and intra‐ or intersexual competition. Therefore, one‐sex‐only, or pooled‐sex approaches might provide an incomplete understanding of phenotypic patterns and the underlying evolutionary drivers (Culumber & Tobler, 2017; Pincheira‐Donoso et al., 2009).

In certain cases, environmental conditions change over short geographic distances, providing an excellent setup to study adaptive evolution on a population or species level (Higginson et al., 2005; Jiang et al., 2019). Classic examples for such environments are mountainous regions with their altitudinal gradients, or deep‐sea environments with their light and resource gradients (Etter et al., 2005; Qu et al., 2014; Steinbauer et al., 2013). Albeit often neglected, surface *vs*. subterranean environments are also examples of such study systems. Surface habitats differ markedly from subterranean ones in many aspects, such as the presence of light, higher degree of daily and seasonal environmental fluctuations (Lauritzen, 2018), increased food availability (Culver & Pipan, 2019), more diverse communities (Gibert & Deharveng, 2002), which can result in higher risk of predation and increased interspecific competition (Manenti et al., 2023; Sket, 2008). The transition from surface to subterranean environment takes place over a distance from some meters to some tens of meters.

Organisms inhabiting subterranean environments commonly show similarities in morphology (Fišer, 2019a), life‐history (Venarsky et al., 2023), physiology (Di Lorenzo et al., 2023) and behaviour (Kowalko, 2019), collectively referred to as troglomorphism (Christiansen, 1962, 1965). Troglomorphies were presumably acquired after the colonisation of the subterranean environment (Ribera et al., 2018), and allegedly impede (re)colonisation of the surface habitats (Horváth et al., 2023; Langille et al., 2022). However, there are documented examples of secondary occurrence of troglomorphic taxa at the surface‐subterranean boundary, including the scorpion family *Typhlochactidae* mitchel, 1971 (Prendini et al., 2010), the isopod *Monolistra pavani* arcangeli, 1942 (Manenti et al., 2022) or certain species of the amphipod *Niphargus* schiödte, 1849 (Copilaş‐Ciocianu et al., 2018; Fišer et al., 2006, 2014; Pérez‐Moreno et al., 2017). These species that are primarily adapted to the subterranean environment are more active during the night in surface or ecotone habitats (Manenti & Barzaghi, 2021), probably to avoid exposure to UV radiation (Langecker, 2000) or visual predators (Clark et al., 2003; Culp & Scrimgeour, 1993). They express photophobic behaviour, a presumed habitat choice mechanism preventing them to move beyond the boundary of the habitat these species are adapted to (Fišer et al., 2016; Simčič & Brancelj, 2007; Wang et al., 2023). Despite multiple cases of species that secondarily appear at the surface‐subterranean boundary (Borko et al., 2021), no study so far explored the phenotypic changes that emerge with the transition from the subterranean environment to the surface‐subterranean boundary.

In this study, we fill this gap using amphipods of the *Niphargus* genus. The genus diversified in the subterranean environment (interstitial habitats), but certain species secondarily colonised springs (Borko et al., 2021; Copilaş‐Ciocianu et al., 2017, 2018; Fišer et al., 2006; Luštrik et al., 2011). Springs are ecotones between surface and subterranean environments (Cantonati et al., 2006). All *Niphargus* species show some level of troglomorphy (lack of pigmentation, and degeneration of eyes; Fišer, 2019b). Many species are negatively phototactic (Borowsky, 2011; Fišer et al., 2016) and express functional visual opsins (Pérez‐Moreno et al., 2018) that likely play a role in habitat choice and survival in the ecotone zone (Fišer et al., 2016). Moreover, species from springs have smaller eggs than species from subterranean habitats (Fišer et al., 2013) and equal sex ratio (female‐biased sex ratio in species from cave lakes; Premate, Borko, Kralj‐Fišer et al., 2021). Somewhat surprisingly, no study to date explored whether species that are able to maintain stable populations in ecotones differ morphologically from species with only subterranean populations.

The aim of this study is to explore whether there are signs of divergence in morphology between subterranean and surface‐subterranean ecotone *Niphargus* and convergence within the habitats. We scrutinised eight *Niphargus* species, four from cave streams and four that have stable populations in ecotones (springs or seepage springs) (Fišer et al., 2006, 2014). Altogether, we analysed 13 functional morphological traits linked to feeding, grooming, locomotion, sensing, and oxygenation of the brood (in case of females) and the gills (both sexes). Although we could not predict the direction of change, we expected that species from caves and ecotones differ in traits related to trophic biology, diurnal activity, and metabolism. Species present in ecotones can access more resources than cave ones, which should be reflected in morphology of feeding structures. The presence of predators in ecotones requires faster swimming to escape, but also better ability to hide. Given that the strength of sexual and fecundity selection varies between the habitats (Balázs et al., 2021), we explore whether the expected habitat divergence was sex‐specific; therefore, we examined individuals from both sexes.

## 2 MATERIALS AND METHODS

### 2.1 Studied species and traits

We sampled eight species from the amphipod genus *Niphargus* (Table 1). Four inhabit subterranean habitats, namely cave streams (*Niphargus stygius* (schiödte, 1847); *Niphargus podpecanus* s. karaman, 1952; *Niphargus spoeckeri* schellenberg, 1933; *Niphargus scopicauda* fišer, 2010), while four predominantly occur in surface‐subterranean ecotones (Borko et al., 2021) (*Niphargus timavi* s. karaman, 1954; *Niphargus sphagnicolus* rejic, 1956; *Niphargus krameri* schellenberg, 1935; *Niphargus spinulifemur* s. karaman, 1954). In the case of the latter four species, only populations occupying ecotones were sampled. The chosen eight species all evolved from subterranean ancestors and some of them went through a shallow subterranean phase according to previous comprehensive studies (Borko et al., 2021; Copilaş‐Ciocianu et al., 2018). The chosen species show no habitat‐based phylogenetic separation (Borko et al., 2021). Previous studies showed that water current is a potent selection force that can override other putative selection agents, such as competition (Delić et al., 2016). To account for confounding effects of water currents, all herein studied species live in streams and belong the ‘cave stream’ morphotype, representing large to medium‐sized species with relatively short appendages (Copilaş‐Ciocianu et al., 2018; Trontelj et al., 2012).

**TABLE 1:** The eight studied *Niphargus* species and their sample sizes.

Species name	Habitat	Male	Female	Total	Date of collection	Place of collection
*Niphargus stygius*	Cave	32	28	60	17.1.	Unška Koliševka, Unec, Slovenia
*Niphargus podpecanus*	Cave	25	29	54	14.1.	Jama v Peklu, Kočevje, Slovenia
*Niphargus spoeckeri*	Cave	28	18	46	17.1.	Planinska jama, Pivka, Planina, Slovenia
*Niphargus scopicauda*	Cave	24	36	60	14.1.	Belojača, Makole, Slovenia
*Niphargus timavi*	Ecotone	31	25	56	14.1.	Kolaški potok, Zabiče, Slovenia
*Niphargus sphagnicolus*	Ecotone	24	36	60	17.1.	Mostec, Rožnik, Ljubljana, Slovenia
*Niphargus krameri*	Ecotone	40	30	70	14.1.	Spring, Brhaji, Grdoselo, Pazin, Croatia
*Niphargus spinulifemur*	Ecotone	40	20	60	14.1.	Stream, under the bridge, Predloka, Dekani, Slovenia
Total		244	222	466		

### 2.2 Sampling, slide preparation and measurements

**FIGURE 1:**
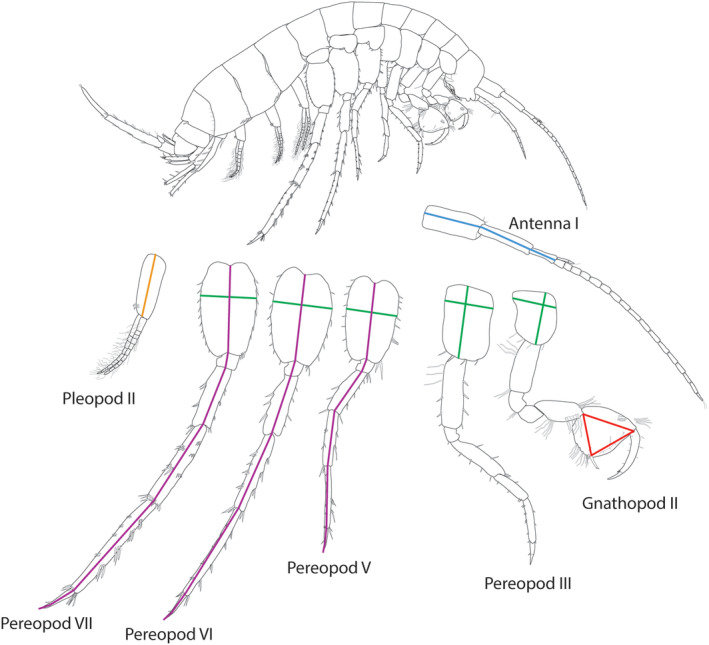
A general drawing of *Niphargus* with the measured traits. Measurements are indicated by coloured lines. The colours coincide with the functional groups of the traits: Blue: Chemo‐ and mechano sensing, red: Feeding and grooming, green: Ventral channel traits related to oxygenation of brood and gills and swimming, purple: Crawling locomotion and sensing, orange: Swimming and oxygenation.

All animals were collected on the 14th and 17th of January 2020 and brought to the University of Ljubljana. Samples were preserved in 96% ethanol. To minimise the distraction of our data by allometric growth, only non‐juvenile individuals were collected based on the presence of marsupium in case of females and penis in case of males. Details of sampling sites are available in Table 1. Altogether 466 (cave female = 111, cave male = 109, ecotone female = 111, ecotone male = 135) individuals were sampled (Table 1). Antenna I, gnathopod II, pereopod III‐VII and pleopod II of each specimen were dissected from the right side. Then all the aforementioned appendages were mounted on regular glass slides (VWR International) in Kaiser's glycerine gelatine (Merck KgaA) and were covered with a coverslip. Two whole body images of the animals were taken after dissecting the appendages from one side with a Canon 600D camera (Canon Inc.). Measurements of body length were performed using Tps Utility v.1.78 (Rohlf, 2019) and Tps Dig2 v.2.30 software (Rohlf, 2017), while slide‐mounted appendages (Figure 1) were measured using a Zeiss Axioscope II microscope and ImageFocus Alpha (Euromex) program package. All measurements were carried out by the same person (A.B.) following the guidelines and landmarks provided by Fišer et al. (2009).

### 2.3 Studies traits

The 13 measured functional morphological traits are depicted in Figure 1. Total body length was measured as a proxy for body size. It was only measured to generate body size corrected values of the measured traits. *Antenna I* was measured as a proxy for sensory function (Fišer et al., 2017). In most crustaceans this is a primary sensory appendage (Schmidt & Mellon, 2010). It bears chemical and mechanical sensory structures (Boxshall, 2004), which are particularly important to compensate for the loss of vision. We found that the flagellum is broken distantly in a large proportion of our samples. To understand whether peduncle length predicts the length of antenna I, we measured five randomly selected individuals with visibly intact flagella from each sex in each population. As peduncle length highly correlated with both flagellum (*r* = 0.86 [95% CI: 0.78–0.91]; *t*_78_ = 124.616; *p* < .001) and total antenna length (*r* = 0.93 [95% CI: 0.9–0.96]; *t*_78_ = 22.893; *p* < .001) of the antenna I, we used the peduncle length as a proxy for antenna I length in our study.

The *gnathopod II propodus* was used as a proxy of trophic position. The propodi are used for feeding and grooming and their size shows positive correlation with trophic position and food availability; as it was shown not only in case of the *Niphargus* genus (Premate, Borko, Delić et al., 2021) but in several other amphipod families such as Hyalellidae, Lysianassidae and Caprellidae too (Caine, 1974; Cothran & Jeyasingh, 2010; Legeżyńska et al., 2012). Three traits of the gnathopod II propodus were measured as a proxy for size: length, width, and diagonal. We used the added‐up value of these three measures to quantify the size of the gnathopod II propodus (Fišer et al., 2009).

Estimation of the *depth and closedness of ventral channel* was used as a proxy of oxygen exchange. Amphipods use self‐generated current flowing through the ventral channel to oxygenate gills (Sutcliffe, 1984). The flow also brings chemical cues towards the anterior chemoreceptors, food particles towards the mouthparts in case of filter‐feeders and aids jet propulsion, which is important for swimming (Dahl, 1978; Fišer, 2019b; Trontelj et al., 2012). Certain parts of the ventral channel (coxal plate of gnathopod II and pereopod III) are also related to marsupium size and aeration of the brood in females (Fišer et al., 2013). To quantify the depth and closedness of the ventral channel, we measured the coxal plate depth and width of the gnathopod II, pereopod III and the width of the basis of pereopods V–VII (hereafter ventral channel traits).

*Pleopod and pereopod lengths* were used to assess locomotor capacities. Beating of pleopods in amphipods generates water current through the ventral channel and jet propulsion (Boudrias, 2002; Trontelj et al., 2012), thus aids swimming and oxygenation of the brood and the gills (Sullivan & Herberholz, 2013). To quantify pleopod size, the peduncle length of pleopod II was measured (hereafter pleopod II). Pereopod V‐VII total length was measured due to its (crawling) locomotor and sensory function (Kralj‐Fišer et al., 2020; Steele, 1988; Trontelj et al., 2012).

### 2.4 Statistical analyses

First, we corrected the data for body size by calculating residuals for all traits from linear regressions against body size. Next, we ran a multivariate linear model (mLM) including all body size corrected traits as dependent variables, and species, sex, and the species × sex interaction as explanatory variables. As all explanatory variables were significant (see 3. Results), we then continued with trait‐by‐trait generalised least square (GLS) models, with the same explanatory variable structure as the mLM. Model selection for each GLS model was performed according to Zuur et al. (2009). We chose the best explanatory variable structure by comparing AIC values. The full model performed best for all traits. As graphical inspection of the data indicated heterogeneous variances, we allowed the variances to differ between groups with VarIdent function with species × sex interaction in all models, except gnathopod II propodus, where instead of the interaction only species was used. To test for habitat effects and sex effects within habitats, we ran pre‐planned comparisons (linear contrasts) (Ruxton & Beauchamp, 2008). We tested whether (i) measured traits differ between the habitats, (ii) sexual dimorphism is present in the measured traits within the cave or within the ecotone habitat, (iii) measured traits of cave females differ from ecotone females and measured traits of cave males differ from ecotone males and (iv) the extent of sexual dimorphism differs between the habitats. We applied GLS models followed by pre‐planned comparisons instead of building linear mixed effect models with habitat as a main effect and species within habitat as a random effect because the low number of levels in the random effect results in low degrees of freedom and inflate the chance for Type I error (Arnqvist, 2020; Harrison et al., 2018). To understand whether the extent of sexual dimorphism differs between the habitats (hereafter habitat dependent sexual dimorphism) we compared the results on sexual dimorphism within habitat type using contrast. The number of species used in the study was low for phylogenetic correction, therefore we did not correct for phylogenetic non‐independence (Kralj‐Fišer et al., 2020). However, previous phylogenetic analyses shows that the species used in this study that occur within the same habitat type are not closely related but represent various phylogenetic lineages (e.g. Borko et al., 2021) implying no habitat‐based phylogenetic separation, but repeated appearance of species that are able to colonise ecotones. For the GLS models, we used *nlme* v.3.1–152 package (Pinheiro et al., 2021). Linear contrasts were calculated with *emmeans* v.1.7.0 (Lenth, 2020). Due to the large number of tests, we applied Bonferroni correction on the *p*‐values, since this correction method is considered highly conservative. All analyses were carried out using R v. 4.2.1 (R Core Team, 2022).

## 3 RESULTS

All explanatory variables of the mLM were statistically significant (species: Pillai's Trace >0.999; *p* < .001; sex: Pillai's Trace = 0.343; *p* < .001; species × sex: Pillai's Trace = 0.137; *p* < .001). In the GLSs, we found statistically significant main effect of species (all χ^2^ > 1061.61; all *p* < .001) and species × sex interaction (all χ^2^ > 62.58; all *p* ≤ .005) for all traits. The main effect of sex was statistically significant for all traits (all χ^2^ > 634.25; all *p* < .001) except antenna I (χ^2^ = 0.16; *p* = .68) and gnathopod II propodus (χ^2^ = 3.31; *p* = .07) (Table 2).

**TABLE 2:** Results of the sex main effect of the generalised least square (GLS) model and the pre‐planned comparisons.

Trait	Sex		Habitat		Sexual dimorphism within habitat				Habitat divergence within sex				Habitat dependent sexual dimorphism	
	F–M		C–E		CF–CM		EF–EM		CF–EF		CM–EM		CF–CM vs. EF–EM	
	χ^2^	Pr(>χ^2^)	t.ratio	*p*	t.ratio	*p*	t.ratio	*p*	t.ratio	*p*	t.ratio	*p*	t.ratio	*p*
Antenna I^1^	**X**		**X**		**X**		**X**		**CF < EF**		**X**		**C > E**	
	0.164	0.685	−1.603	.657	−2.427	.094	2.264	.145	−**3.889**	**<.001**	1.104	1.0	−**3,314**	**.005**
Gnathopod II size^2^	**X**		**C > E**		**X**		**EF > EM**		**CF > EF**		**CM > EM**		**C < E**	
	3.305	0.069	**16.516**	**<.001**	−0.640	1.0	**3.860**	**<.001**	**9.351**	**<.001**	**14.189**	**<.001**	−**2.838**	**.028**
Coxa II width^3^	**F > M**		**X**		**CF > CM**		**EF > EM**		**X**		**X**		**X**	
	**104.477**	**<0.001**	−1.429	.923	**5.941**	**<.001**	**8.459**	**<.001**	−2.359	.113	0.431	1.0	−2.017	.266
Coxa II depth^3^	**F > M**		**C < E**		**CF > CM**		**EF > EM**		**CF < EF**		**X**		**X**	
	**499.175**	**<0.001**	−**4.561**	**<.001**	**14.374**	**<.001**	**17.266**	**<.001**	−**4.628**	**<.001**	−1.930	.326	−1.73	.506
Coxa III width^3^	**F > M**		**C < E**		**CF > CM**		**EF > EM**		**CF < EF**		**CM < EM**		**X**	
	**84.924**	**<0.001**	−**4.077**	**<.001**	**5.475**	**<.001**	**7.792**	**<.001**	−**3.079**	**.013**	−**2.691**	**.044**	−0.864	1.0
Coxa III depth^3^	**F > M**		**X**		**CF > CM**		**EF > EM**		**X**		**X**		**X**	
	**634.247**	**<0.001**	0.040	1.0	**14.731**	**<.001**	**22.075**	**<.001**	−1.336	1.0	1.401	.971	−1.936	.321
Pereopod V base^3^	**F > M**		**C < E**		**CF > CM**		**EF > EM**		**CF < EF**		**X**		**C < E**	
	**130.957**	**<0.001**	−**6.575**	**<.001**	**5.09**	**<.001**	**11.184**	**<.001**	−**8.329**	**<.001**	−1.589	.113	−**4.142**	**<.001**
Pereopod VI base^3^	**F > M**		**C < E**		**CF > CM**		**EF > EM**		**CF < EF**		**X**		**C < E**	
	**146.444**	**<0.001**	−**6.738**	**<.001**	**6.078**	**<.001**	**11.344**	**<.001**	−**8.170**	**<.001**	−2.301	.131	−**3.055**	**.014**
Pereopod VII base^3^	**F > M**		**C < E**		**CF > CM**		**EF > EM**		**CF < EF**		**CM < EM**		**X**	
	**182.275**	**<0.001**	−**8.434**	**<.001**	**7.265**	**<.001**	**12.31**	**<.001**	−**8.932**	**<.001**	−**3.787**	**<.001**	−2.417	.096
Pereopod V length^4^	**F > M**		**C > E**		**CF > CM**		**EF > EM**		**CF > EF**		**CM > EM**		**C < E**	
	**126.829**	**<0.001**	**17.336**	**<.001**	**5.338**	**<.001**	**11.365**	**<.001**	**9.825**	**<.001**	**14.906**	**<.001**	−**2.917**	**.022**
Pereopod VI length^4^	**F > M**		**C > E**		**CF > CM**		**EF > EM**		**CF > EF**		**CM > EM**		**C < E**	
	**104.381**	**<0.001**	**22.026**	**<.001**	**4.354**	**<.001**	**11.339**	**<.001**	**13.108**	**<.001**	**18.126**	**<.001**	−**3.195**	**.008**
Pereopod VII length^4^	**F > M**		**C > E**		**CF > CM**		**EF > EM**		**CF > EF**		**CM > EM**		**C < E**	
	**73.456**	**<0.001**	**21.755**	**<.001**	**3.012**	**.016**	**9.674**	**<.001**	**12.769**	**<.001**	**17.918**	**<.001**	−**3.955**	**<.001**
Pleopod II^5^	**F < M**		**X**		**CF < CM**		**EF <EM**		**CF < EF**		**X**		**C > E**	
	**42.128**	**<0.0014**	−1.584	.683	−**6.102**	**<.001**	−**2.865**	**.026**	−**3.029**	**.016**	0.831	1	−**2.747**	**0.38**

*Note*: Numbers in upper index next to the trait names represent functional groups: 1: Chemo‐ and mechano‐sensing, 2: Feeding and grooming, 3: Ventral channel traits related to oxygenation of brood and gills and swimming, 4: Crawling and sensing, 5: Swimming and oxygenation. Statistically significant results are bolded.

Abbreviations: C, cave; CF, cave female; CM, cave male; E, ecotone; EF, ecotone female; EM, ecotone male; F, female; M, male; X, non‐significant results.

The results of the pre‐planned comparisons are shown in Table 2 and Figure S1. In the habitat‐based comparisons, gnathopod II propodus (feeding and grooming) and pereopods (crawling locomotion traits) were larger in cave than in ecotone species. Most ventral channel traits (oxygenation, swimming) were larger in ecotones except for the width of coxal plate II and the depth of coxal plate III, where no habitat effect was detected. This translates to deeper and more closed ventral channel in ecotone than in cave species. There were no habitat‐related differences between the species in antenna I (sensing) and pleopod II (water flow generation, jet propulsion and oxygenation).

The comparison of the same sex between the habitats yielded that in females, antenna I is longer, the ventral channel is deeper and more closed (except for the width of coxal plate II and depth of coxal plate III), and pleopod II is longer in ecotone than in cave species. Gnathopod II propodus is larger and pereopods are longer in cave than in ecotone species.

In males, the gnathopod II propodus is larger and the pereopods are longer in cave species than in ecotone species, similarly to females. However, we found no habitat effect in males regarding antenna I, ventral channel traits (except the width of coxa III and basis of pereopod VII showing the same pattern as in females) and pleopod II.

When testing sexual dimorphism within habitats we found that females have deeper and more closed ventral channels than males and longer pereopods, while males have longer pleopod II than females in both habitats. However, in species from ecotones, we noted an additional sexually dimorphic trait, the gnathopod II propodus, which was larger in females.

Comparison of the extent of sexual dimorphism between the habitats showed that sexual dimorphism is more expressed in case of antenna I and pleopod II in caves. In case of ecotones, sexual dimorphism is more expressed in gnathopod II and in two traits related to the ventral channel, namely the bases of pereopods V–VI and the lengths of pereopod V–VII.

## 4 DISCUSSION

The morphological differences along the surface‐subterranean gradient were hitherto studied in the context of colonisation of the subterranean environment. At the same time there are cases when taxa primarily adapted to the subterranean environment thrive in surface‐connected habitats. These reversal processes were noted, but never addressed. Here, by comparing four obligate cave‐dwelling species to four species inhabiting surface‐subterranean ecotones of the *Niphargus* genus that is primarily adapted to the subterranean environment, we detected morphological divergence between the habitats in nine, sexual dimorphism in 11 and habitat‐dependent sexual dimorphism in eight out of the 13 studied morphological traits. We note that in ecotones, neither species re‐evolved the lost structures, such as eyes or pigmentation, supporting Dollo's principle (Recknagel et al., 2023). Below, we first discuss the detected patterns by functional groups of morphological traits and then the overall trends.

### 4.1 Variation in functional traits

*Antenna I* is responsible for chemical and mechanical sensing. We found no marked differentiation between the habitats or the sexes; we only found elongation of this trait in ecotones in females when compared to cave females, which also results in differences in sexual dimorphism between the habitats. Lack of general differentiation between the habitats is not a surprise. All *Niphargus* species lack eyes, therefore species from both habitats rely on the same sensory modalities, making extraoptic senses pivotal. This is in contrast with surface species that colonised caves and went through eye reduction (Jeffery et al., 2000). Sexual dimorphism of the antenna generally denotes differences in mating behaviour. Differences in the antenna between the sexes can aid successful mate searching or help in holding the females in precopulatory mate guarding or during copulation (Boxshall & Jaume, 2013). Although very little is known about mating of *Niphargus*, equal sex ratio in case of the stream morphotype (Premate, Borko, Kralj‐Fišer et al., 2021) and short amplexus (Marin & Palatov, 2019) might not select for sexual differences. At the same time, longer antenna I in ecotones when comparing only females paired with more pronounced sexual dimorphism in caves might be a sign of sex‐dependent environmental selection; however, we cannot explain the pattern based on any sex or habitat‐specific functional benefit.

*Gnathopod II propodus* is responsible for feeding and grooming. We found that cave species generally have larger gnathopod II propodus than ecotone species. The size of this appendage in *Niphargus* covaries with species' trophic position; species with larger gnathopods appear higher in trophic structure of subterranean communities (Premate, Borko, Delić et al., 2021). Trophic selectivity is predicted to vary with resource availability (Correa & Winemiller, 2014; Gong et al., 2023), hence expansion of the trophic niche in food‐deprived environments such as caves seems to be beneficial. Indeed, a comparison of cave and surface‐adapted species of the asellids, *Bragasellus* and *Proasellus* suggested that subterranean species tend to feed less selectively (Francois et al., 2020). Saccó et al. (2022) demonstrated that the cave‐adapted atyid shrimp *Stygiocaris stylifera* holthuis, 1960 can switch between feeding strategies (predator to decomposer) when food availability is changing. Our results corroborate this pattern. While larger gnathopod II in cave species implies that they feed over two trophic levels (Premate, Borko, Delić et al., 2021), smaller gnathopod II in ecotones suggest higher level of specialisation. The latter can be explained by both more abundant food in ecotones and stronger interspecific competition. The larger gnathopod II in females in ecotones and the significant habitat‐dependent sexual dimorphism is probably a result of high sexual dimorphism in the ecotone‐dwelling *N. spinulifemur* (Figure S1). Therefore, we argue that the gnathopod size is not under strong sexual selection in case of most species (Premate, Borko, Delić et al., 2021), but there can be deviations from the general pattern.

*Ventral channel*, assessed from the widths and depths of coxa II–III and bases of pereopod V–VII, is a multifunctional structure that maintains oxygenation of the gills and brood, aids chemical cue and food transport, and jet propulsion. While deeper coxal plates result in a deeper ventral channel, wider coxae and bases of pereopods determine its closedness. In all cases, the ventral channel is better developed in females, most likely due to breeding functions, such as additional protection, and to enhance oxygenation of the brood (Fišer et al., 2015). An early study showed that females from cave lakes have a deeper and more closed ventral channel than ecotone ones (Fišer et al., 2013). Here, we took into account the confounding effects of water currents and came to opposing conclusions, that is, ecotone species have more developed ventral channel, than cave stream species. This can be connected to (i) lower metabolic rate, an adaptation to low amount of food in cave habitats (Malard & Hervant, 1999; but see Guillaume et al., 2020), (ii) more emphasised need to protect the eggs in the marsupium in ecotones due to the higher number of predators or (iii) increased time spent with faster swimming (aided by jet propulsion) instead of crawling due to increased risk of predation in ecotones. Although general habitat differences imply that ecotone species have more developed ventral channel, a closer look suggests that most of these differences can be attributed to the differences between females (except coxa width of pereopod II and coxa depth of pereopod III). Similar trait size between the habitats in males, but larger trait size in ecotones in case of females also results in habitat‐dependent sexual dimorphism in bases of pereopods V and VI. Sexual size dimorphism in *Niphargus* species inhabiting cave‐streams and ecotones is generally male‐biased (Premate et al., 2024). The generally larger body size of males might restrict an increase in ventral channel depth, because a similar ratio to females between body size and ventral channel depth could lead to a large body diameter, which could restrain the exploitation of small crevices (Trontelj et al., 2012).

*Pereopods V–VII* probably aid sensing besides its general locomotor function (Fišer et al., 2009). As many crustaceans (Ache, 1982), *Niphargus* bears extra‐optic sensory structures (e.g. setae) along their appendages (Fišer et al., 2009; Kralj‐Fišer et al., 2020). The number of setae increases along with the length of the appendages to aid mechano‐ and chemo‐sensing. In general, we found that females have longer pereopods than males in both habitats, with ecotone species being more sexually dimorphic. Relatively shorter pereopods of males in both habitats probably stem from generally larger body size of males. A comparable pereopod – body size proportion to females would restrain the use of small crevices and could also increase the risk of injuries in case of strong water currents in males (Delić et al., 2016; Kralj‐Fišer et al., 2020; Trontelj et al., 2012). Another possible explanation is that the generally smaller females switch earlier from growth to reproduction, and thus retain their juvenile proportions, meaning proportionally longer pereopods (as it was found in case of pereopod VII by Fišer et al., 2008). We also detected differences between the habitats translating to shorter pereopods in ecotones in case of both sexes. This difference coincides with the observed patterns in cases when surface species colonise caves (Christiansen, 2012). This phenomenon has two mutually non‐exclusive explanations. First, in caves, where resources are scarcer, longer pereopods, which can bear more sensory setae are under positive selection. More seta decreases the chance of missing a mechanical or chemical cue and thus increases their chances to find food or mates. Second, as it has been previously shown in case of *Asellus aquaticus* (linnaeus, 1758), due to the lower number of predators, individuals of cave‐adapted populations tend to shelter less than surface‐adapted conspecifics (Fišer et al., 2016; Horváth et al., 2021). Increased time spent sheltering in ecotones coupled with less time spent searching for food probably selects for shorter pereopods to ease size restrictions of the shelter. This results in ecotone males having the shortest pereopods compared to their body size, thus leading to habitat‐dependent sexual dimorphism in case of these traits. Therefore, we argue that pereopod length differences are possibly one of the most important divergences between cave and ecotone dwelling species of *Niphargus*.

*Pleopod II*, responsible for generating the current through the ventral channel and for jet propulsion and egg oxygenation (Sullivan & Herberholz, 2013), was longer in males in both habitats. The functional benefit of longer pleopod II peduncle in males is twofold. Firstly, a longer pleopod probably generates stronger currents, which can compensate for the shallower ventral channel of males (see above) and thus aid the oxygenation of the gills. Secondly, it enables faster speed when swimming, which increases mobility, therefore it could again compensate for shallower ventral channel and directly affect the efficiency of mate searching, foraging, and escaping from predators. We also detected that females in ecotones have generally longer pleopods than in caves also resulting in decreased extent of sexual dimorphism in ecotones. In case of *Gammarus pulex* (linnaeus, 1758), Van den Berg et al. (2023) found that light increases the amount of swimming. A possible explanation for longer pleopod II in ecotone females and decreased sexual dimorphism in ecotones could be the higher number of visual predators and more emphasised need for swimming during daytime. Therefore, longer pleopod II in ecotone females is probably due to the increased competition and predation, while no such difference between males might be the result of size restriction preventing further growth. It must be noted that in the case of one species, *N. spinulifemur* (ecotone) sexual dimorphism was showing the opposite pattern meaning that females have longer peduncle (Figure S1), but the reason for such difference is unknown.

### 4.2 General trends and conclusions

Our study involving 472 individuals of eight stream ecomorph species of the *Niphargus* genus revealed divergence between the four obligate cave‐dweller vs. four surface‐subterranean ecotone species in nine and patterns of sexual dimorphism in 11 out of 13 functionally important morphological traits. We found high consistency in both habitat‐divergence within the sexes and sexual dimorphism within habitats in the variation of functional morphology in *Niphargus*. The habitat effect marks convergences in phenotypes that emerged in response to environmental similarities within a habitat and divergences related to different selection factors when comparing the two habitat types. The differences between cave and ecotone species were in line with differences detected when surface species colonise caves. The high number of sexually dimorphic traits supports that there are clearly different selection forces acting on the sexes leading to different optima, suggesting complex interplay among environmental, sexual and fecundity selection. The fact that we detected that habitat‐specific selection can differ between sexes leading to habitat‐dependent sexual dimorphism in several cases despite the expected equal sex ratio in both habitats (Premate, Borko, Delić et al., 2021) further supports the complexity of various selective forces and the interactions between them, resulting in the observed phenotypic variation. Therefore, we strongly recommend that in studies aiming to understand adaptive divergence between habitats, sexual differences are also taken into account, because analyses based on one sex only or on pooled sexes might convey false conclusions.

The trait‐by‐trait analyses also allow us to draw some ecologically relevant conclusions. In ecotones, trophic selectivity is probably higher than in caves due to the ease of food deprivation compared to caves, which is reflected in the differences in feeding structure (Gibert & Deharveng, 2002; Saccó et al., 2019). Traits related to the oxygenation of the brood and the gills are also more developed in ecotones, which might reflect the increase in metabolic rate in ecotones, probably linked to higher resource availability (Poulson, 1963). Appendages related to locomotion also show divergence, suggesting differences in the use of the habitats both regarding locomotion modes and sheltering (Horváth et al., 2021).

It should be noted, that the number of studied species per habitat type was somewhat low, making these conclusions prone to error. A low number of studied species requires a tentative generalisation, as a single species may bias results and mask the general pattern. In this study, the ecotone *N. spinulifemur* showed high levels of sexual dimorphism in some traits, which resulted in significant contrasts suggesting habitat‐dependent sexual dimorphism. Apart from extending the study to additional species, we suggest that the time is ripe to move to experimental evolution studies, including common garden experiments. Cave stream and ecotone *Niphargus* seems to be an appropriate model system for such studies. By short‐ and long‐term laboratory rearing experiments, the ecological versatility, the role of phenotypic plasticity (Bilandžija et al., 2020) and natural selection could be directly tested. Solving the problems related to laboratory rearing of cave dwelling invertebrates (Lukić et al., 2024; Mammola et al., 2021) probably would result in the use of cave species as prime model organisms to test general questions of evolutionary biology.

## AUTHOR CONTRIBUTIONS

**Anna Biró:** Data curation (equal); formal analysis (lead); methodology (equal); writing – original draft (lead); writing – review and editing (equal). **Gergely Balázs:** Data curation (equal); methodology (equal); visualization (lead); writing – review and editing (equal). **Žiga Fišer:** Conceptualization (equal); writing – review and editing (equal). **Cene Fišer:** Conceptualization (equal); funding acquisition (equal); resources (equal); writing – review and editing (equal). **Gergely Horváth:** Formal analysis (equal); methodology (equal); writing – review and editing (equal). **Gábor Herczeg:** Funding acquisition (equal); project administration (lead); resources (equal); supervision (lead); writing – review and editing (equal).

## CONFLICT OF INTEREST STATEMENT

The authors declare no competing interests. All authors have read and agreed to the published version of the manuscript.

## Supporting information


Figure S1:

## Data Availability

The original data and the R code are available on Dryad doi https://doi.org/10.5061/dryad.5x69p8dc0.
