# Supplementary material for: From darkness to twilight: Morphological divergence between cave and surface‐subterranean ecotone Niphargus species
Source: Ecol Evol. 2024 Aug 6;14(8):e70061. doi: 10.1002/ece3.70061 (PMC11301267; doi:10.1002/ece3.70061)
Supplement: Supplementary file 1 — Figure S1: [file ECE3-14-e70061-s001.pdf]

Supplementary Figure 1. Results of the pre-planned comparisons. Estimated marginal means and standard errors for one measured morphological trait are shown. Abbreviations denote the names of the species: Tim, *N. timavi*; Sph, *N. sphagnicolus*; Spi, *N. spinulifemur*; Kra, *N. krameri*; Pod, *N. podpecanus*; Spo, *N. spoeckeri*; Sco, *N. scophicauda*; Sty, *N. stygius*. The dashed lines represent means calculated from the raw body size corrected data. Letters represent significant results of the pre-planned comparisons: a, habitat divergence in females; b, habitat divergence in males c, sexual dimorphism within cave habitat; d, sexual dimorphism within ecotone habitat,: *H*, general habitat divergence; *Hsd*, habitat-dependent sexual dimorphism.

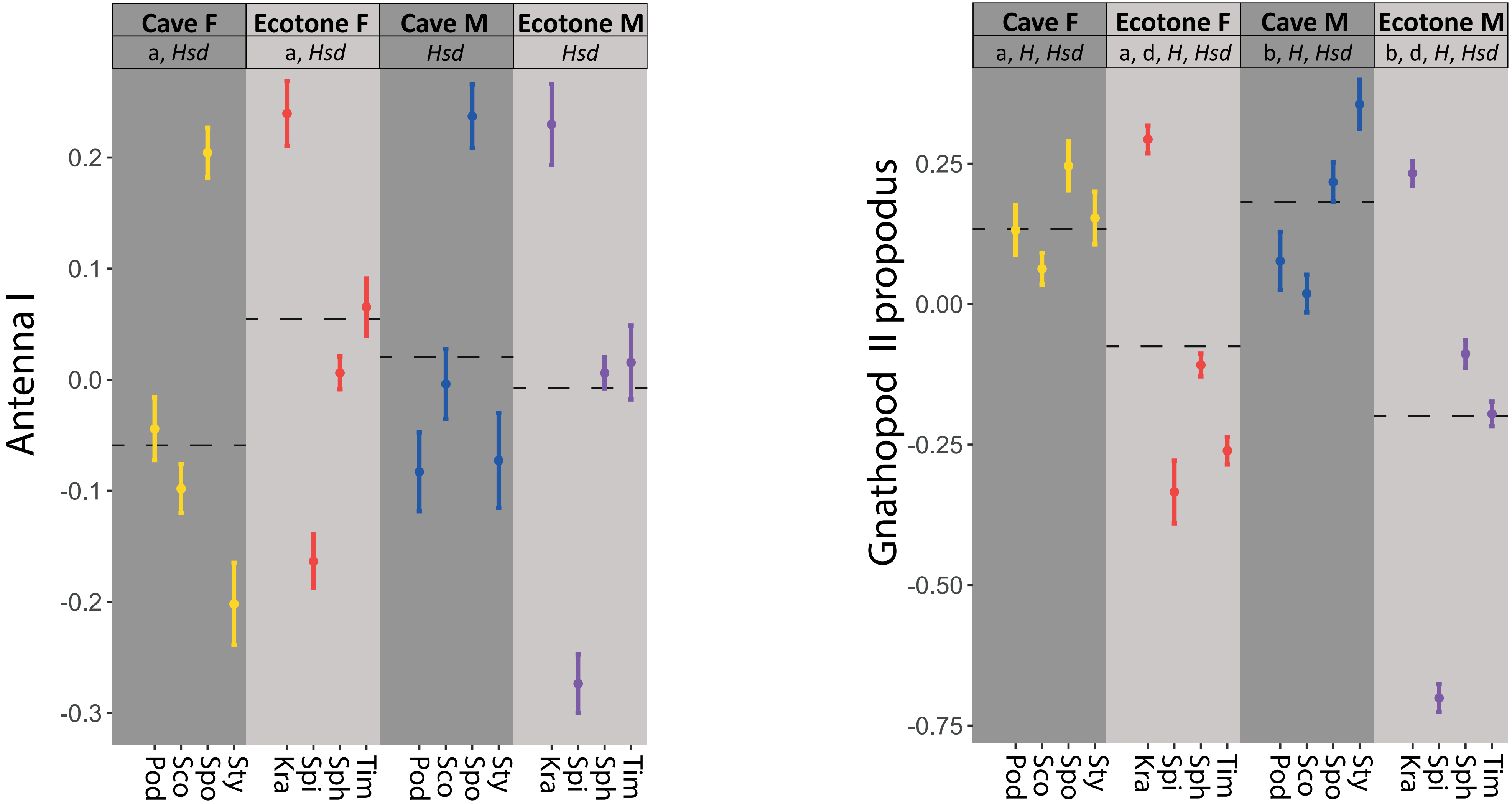

Antenna I is connected to chemo-and mechanosensing. Habitat divergence is significant in case of females (a). The extent of sexual dimorphism differs between the two habitat types (*Hsd*).

Gnathopod II propodus is connected to feeding and grooming. Habitat divergence is significant in case of females (a) and males (b). Sexual dimorphism is significant within the ecotone habitat (d). Shows general habitat divergence (*H*). The extent of sexual dimorphism differs between the two habitat types (*Hsd*).

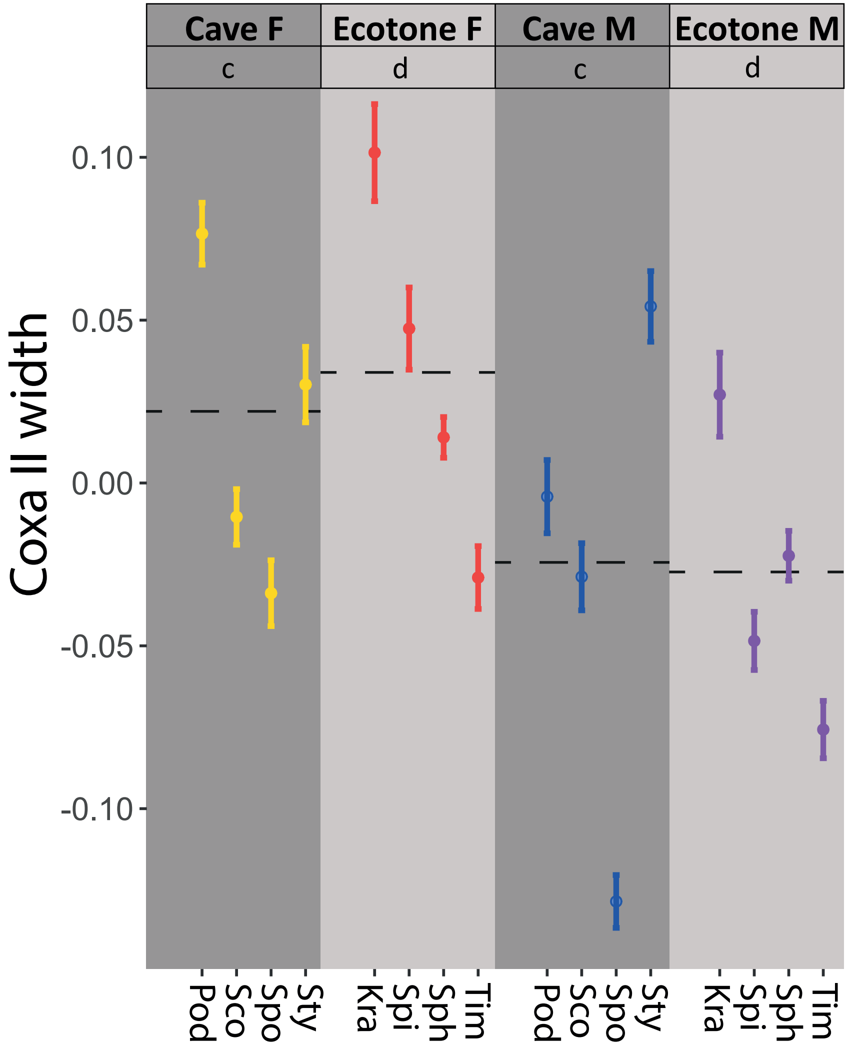

Coxa II width is connected to the ventral channel and have a role in the oxygenation of the brood and the gills and in swimming. Sexual dimorphism is significant both within the cave (c) and within the ecotone habitat (d).

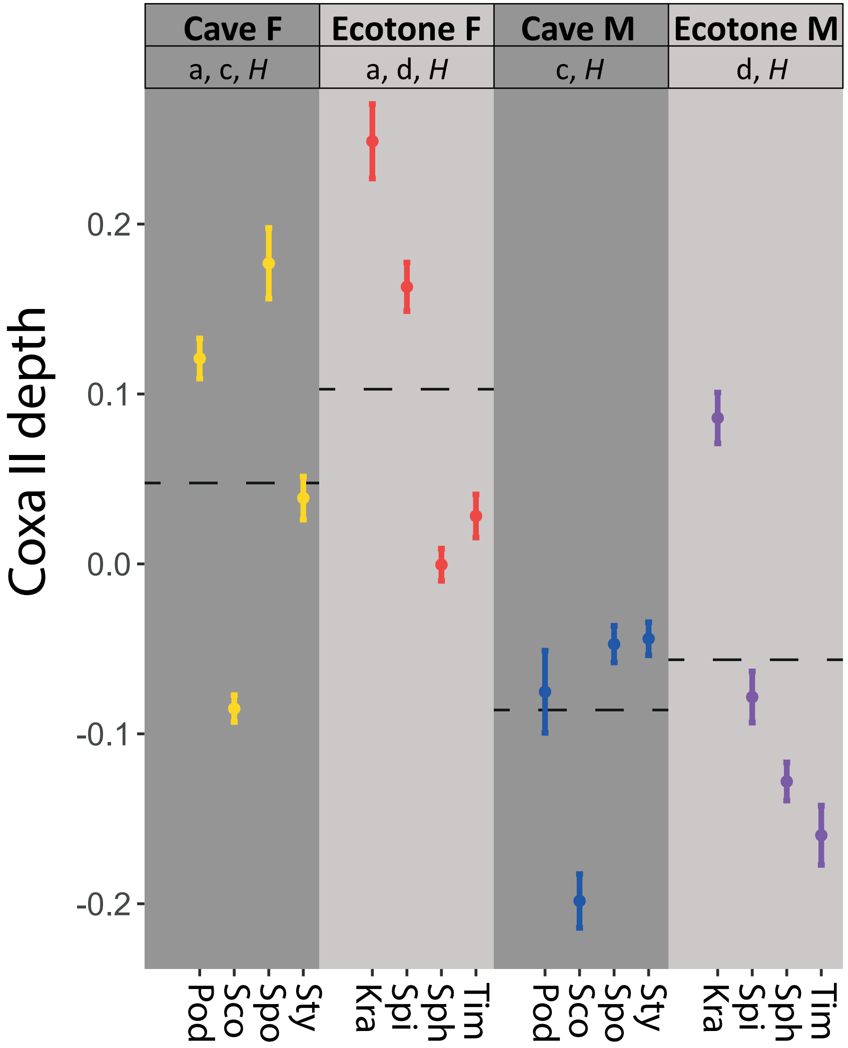

Coxa II depth is connected to the ventral channel and have a role in the oxygenation of the brood and the gills and in swimming. Habitat divergence is significant in case of females (a). Sexual dimorphism is significant both within the cave (c) and within the ecotone habitat (d). Shows general habitat divergence (*H*).

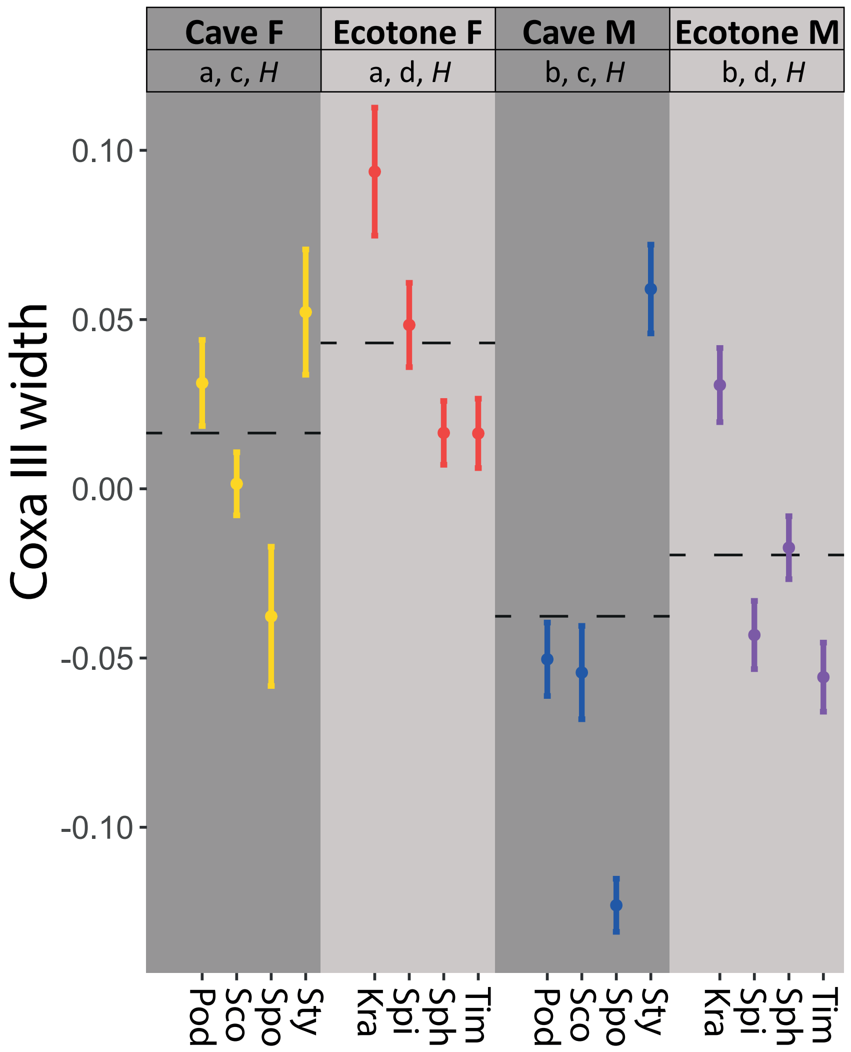

Coxa III width is connected to the ventral channel and have a role in the oxygenation of the brood and the gills and in swimming. Habitat divergence is significant in case of females (a) and males (b). Sexual dimorphism is significant both within the cave (c) and within the ecotone habitat (d). Shows general habitat divergence (*H*).

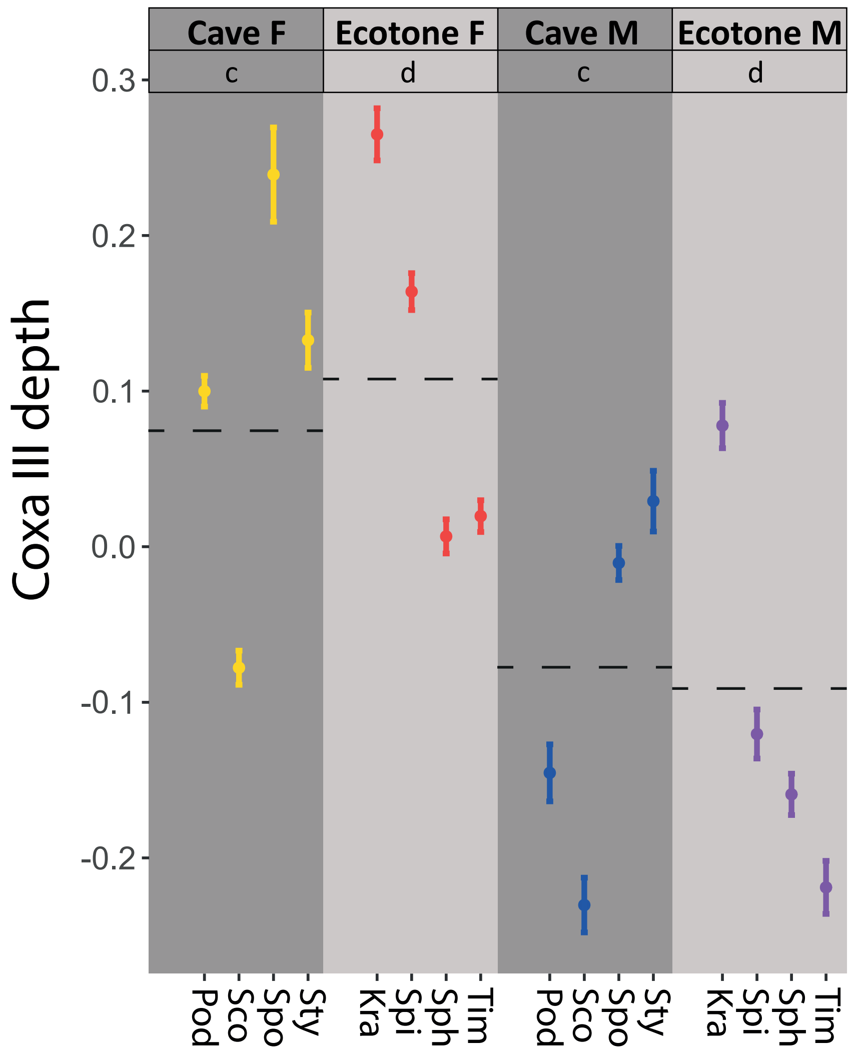

Coxa III depth is connected to the ventral channel and have a role in the oxygenation of the brood and the gills and in swimming. Sexual dimorphism is significant both within the cave (c) and within the ecotone habitat (d).

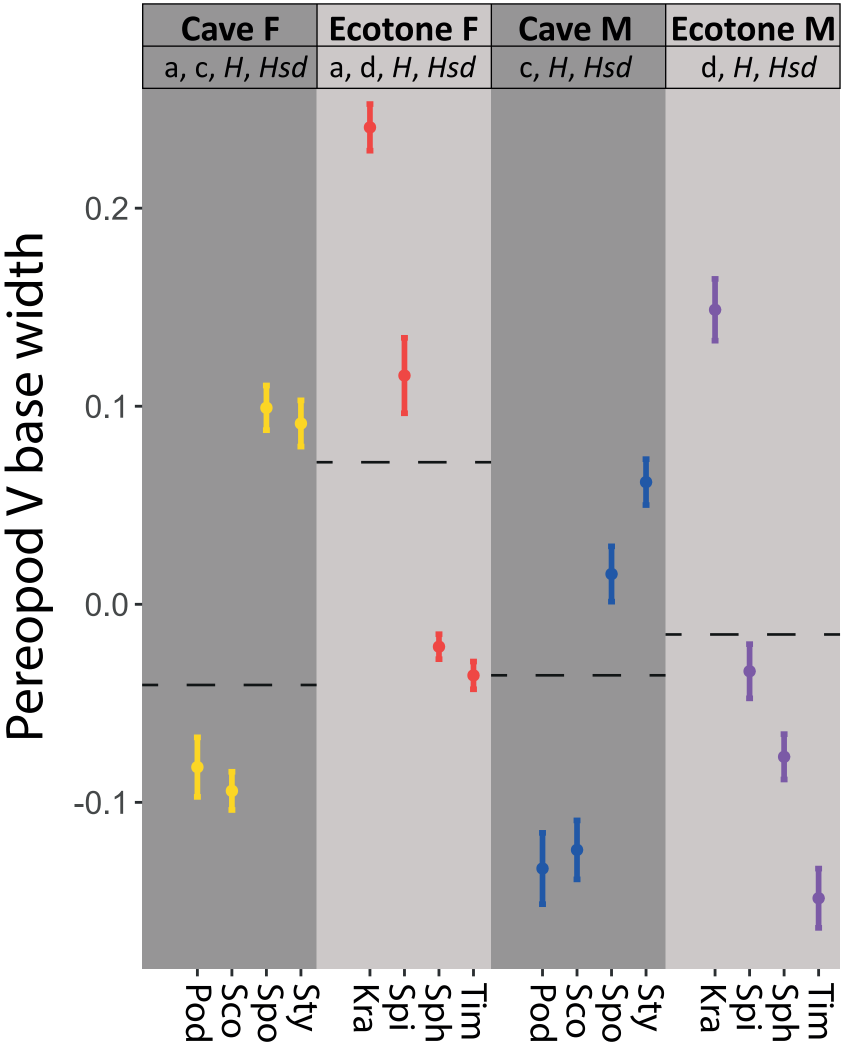

Pereopod V base is connected to of the ventral channel and have a role in the oxygenation of the brood and the gills and in swimming. Habitat divergence is significant in case of females (a). Sexual dimorphism is significant both within the cave (c) and within the ecotone habitat (d). Shows general habitat divergence (*H*). The extent of sexual dimorphism differs between the two habitat types (*Hsd*).

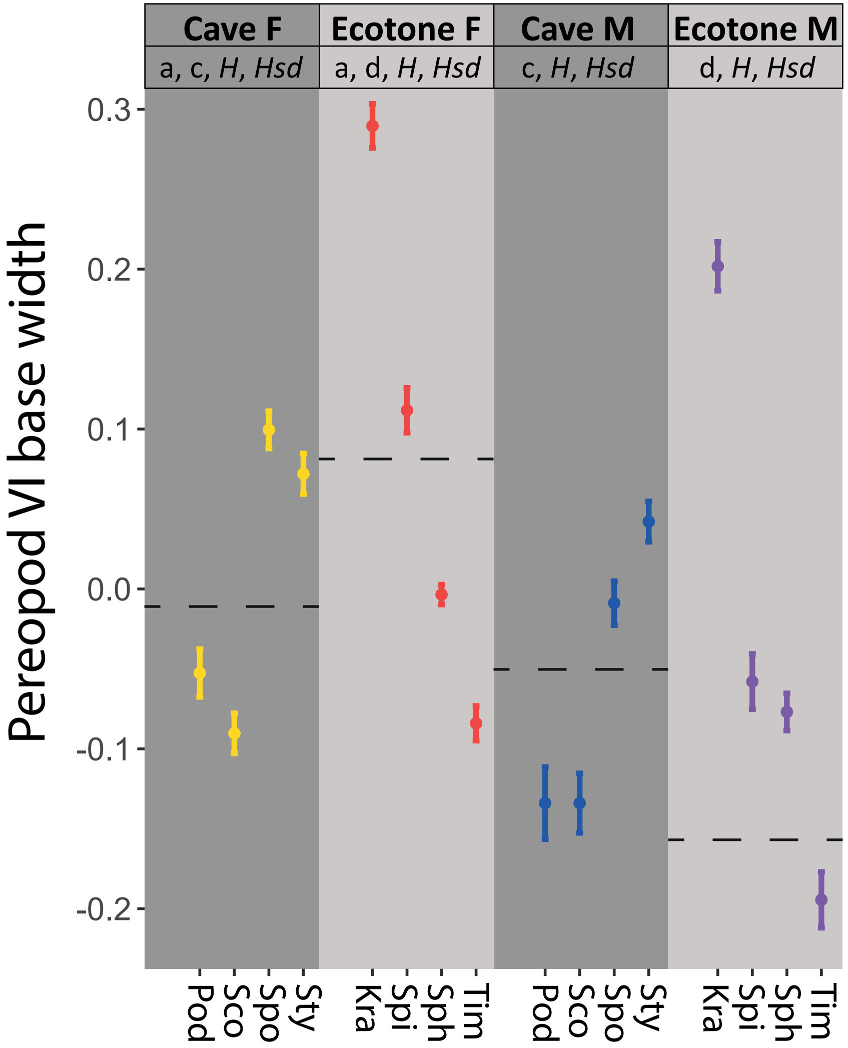

Pereopod VI base is connected to the ventral channel and have a role in the oxygenation of the brood and the gills and in swimming. Habitat divergence is significant in case of females (a). Sexual dimorphism is significant both within the cave (c) and within the ecotone habitat (d). Shows general habitat divergence (*H*). The extent of sexual dimorphism differs between the two habitat types (*Hsd*).

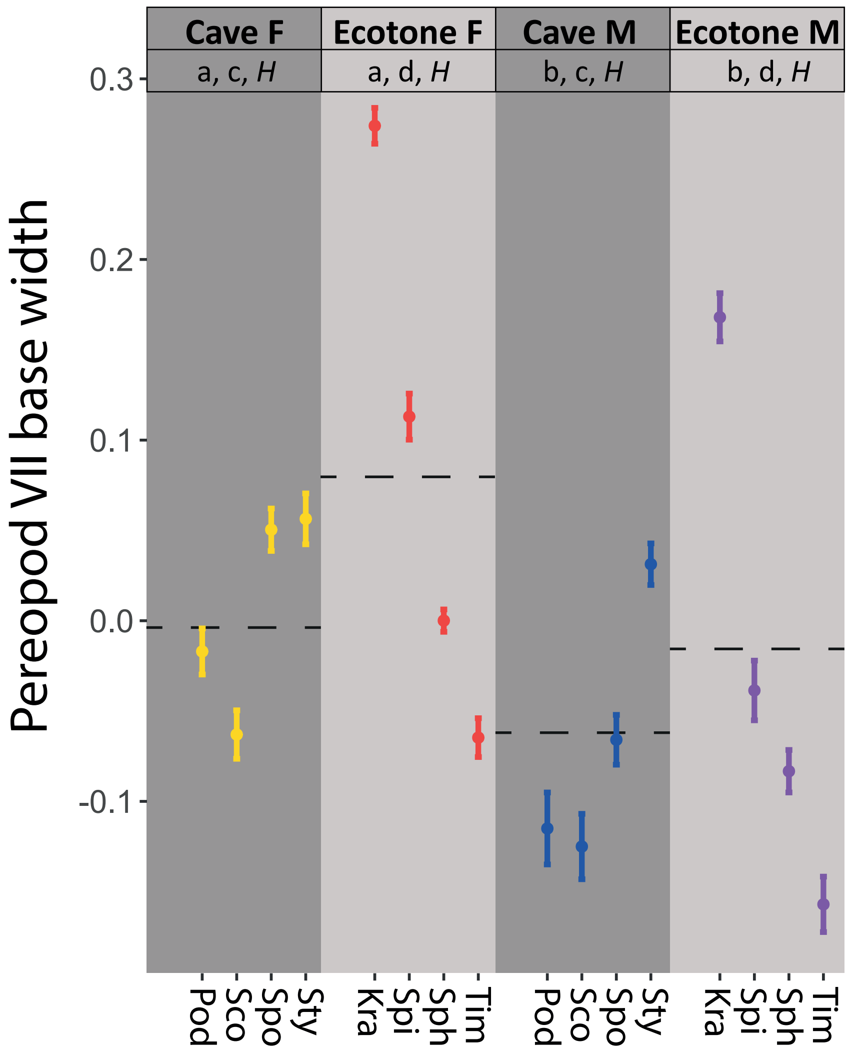

Pereopod VII base is connected to the ventral channel and have a role in the oxygenation of the brood and the gills and in swimming. Habitat divergence is significant in case of females (a) and males (b). Sexual dimorphism is significant both within the cave (c) and within the ecotone habitat (d). Shows general habitat divergence (*H*).

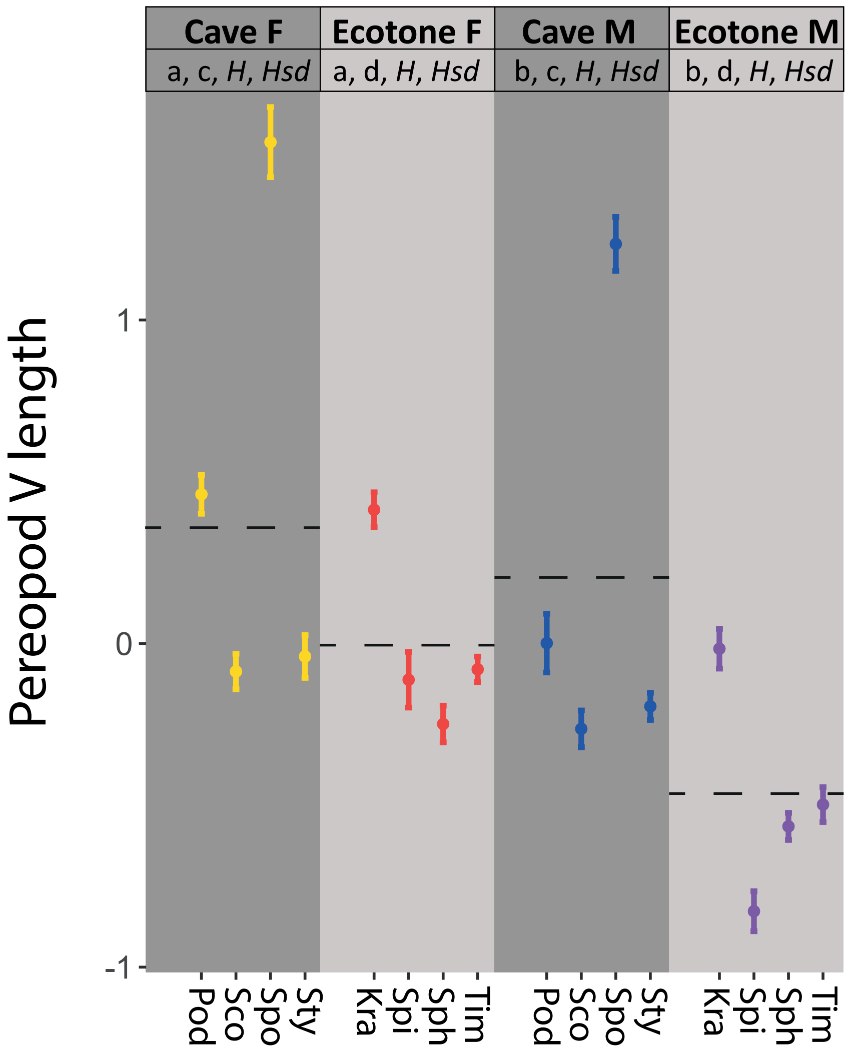

Pereopod V length is connected to crawling locomotion and sensing. Habitat divergence is significant in case of females (a) and males (b). Sexual dimorphism is significant both within the cave (c) and within the ecotone habitat (d). Shows general habitat divergence (*H*). The extent of sexual dimorphism differs between the two habitat types (*Hsd*).

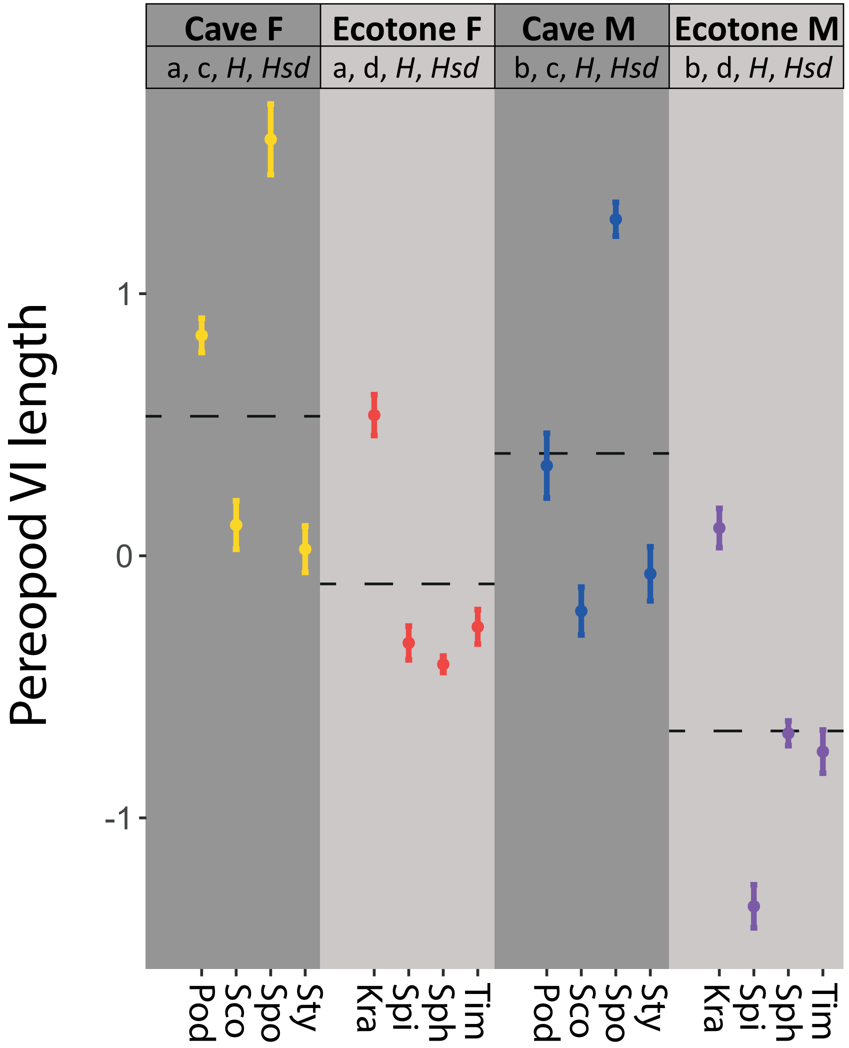

Pereopod VI length is connected to crawling locomotion and sensing. Habitat divergence is significant in case of females (a) and males (b). Sexual dimorphism is significant both within the cave (c) and within the ecotone habitat (d). Shows general habitat divergence (*H*). The extent of sexual dimorphism differs between the two habitat types (*Hsd*).

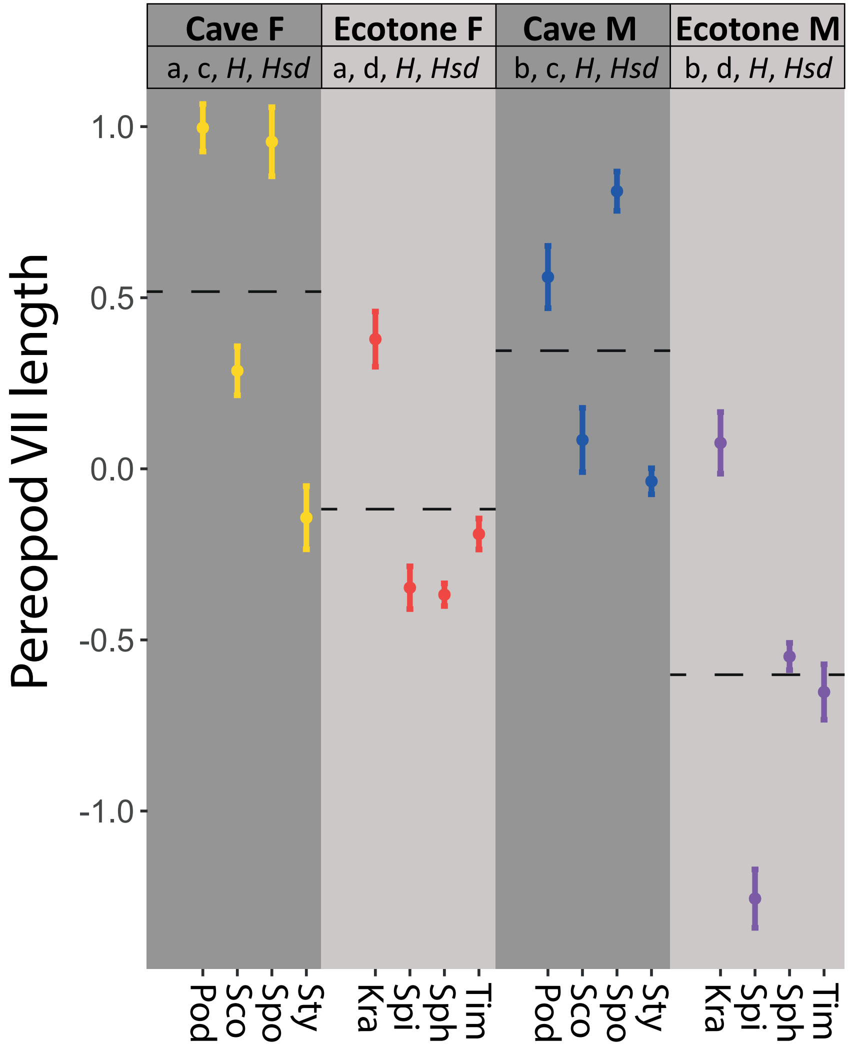

Pereopod VII length is connected to crawling locomotion and sensing. Habitat divergence is significant in case of females (a) and males (b). Sexual dimorphism is significant both within the cave (c) and within the ecotone habitat (d). Shows general habitat divergence (*H*). The extent of sexual dimorphism differs between the two habitat types (*Hsd*).

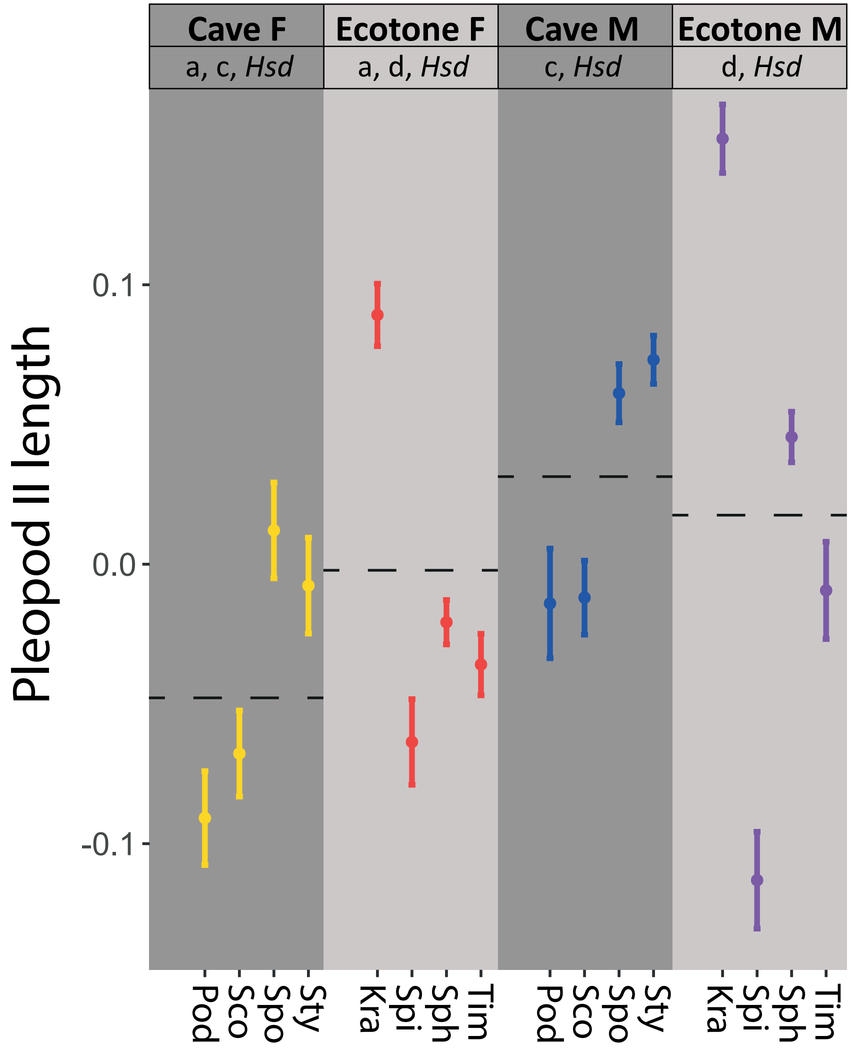

Pleopod II is connected to swimming and oxygenation. Habitat divergence is significant in case of females (a). Sexual dimorphism is significant both within the cave (c) and within the ecotone habitat (d). The extent of sexual dimorphism differs between the two habitat types (*Hsd*).
